# Global dataset combining open-source hydropower plant and reservoir data

**DOI:** 10.1038/s41597-025-04975-0

**Published:** 2025-04-17

**Authors:** Jignesh Shah, Jing Hu, Oreane Y. Edelenbosch, Michelle T. H. van Vliet

**Affiliations:** 1 Department of Physical Geography, Utrecht University, PO Box 80.115, 3508, TC Utrecht, the Netherlands; 2 Copernicus Institute of Sustainable Development, Utrecht University, PO Box 80.115, 3508, TC Utrecht, the Netherlands; 3 PBL Netherlands Environmental Assessment Agency, Den Haag, the Netherlands

**Keywords:** Energy supply and demand, Hydrology

## Abstract

Hydropower is a crucial renewable source that depends heavily on water availability. Analyzing drought and climate change impacts on hydropower potential requires detailed data on both hydropower plant attributes (e.g. plant type and head) and reservoir characteristics (e.g. area, depth and volume). However, existing open-source datasets are poorly integrated: hydropower plant datasets often lack reservoir information, while reservoir datasets commonly miss hydropower plant information. This paper addresses this gap by introducing GloHydroRes, a global dataset that combines existing open-source hydropower plant and reservoir datasets. GloHydroRes includes attributes like plant location, head, plant type as well as reservoir details such as dam and reservoir location, dam height, reservoir depth, area, and volume for 7,775 plants in 128 countries. GloHydroRes covers nearly 79% and 81% of the global installed capacity when compared with installed hydropower data as reported by the EIA(2022) and IRENA (2023), respectively. The open-source GloHydroRes dataset provides crucial data to improve hydropower generation modelling at plant level and can support energy security and planning at continent to global scale.

## Background & Summary

Hydropower is considered an important source of renewable energy due to its flexibility and storage capabilities, accounting for nearly 14% of all electricity generation in 2023^1^. Furthermore, to decarbonize the power sector and achieve the Paris Agreement targets, hydropower capacity needs to be expanded^2^. This is particularly true in developing countries where growing population and economic development increase energy demand^3,4^. These countries also coincidentally tend to have high exploitable hydropower potential^5^. Hydropower potential mainly depends on water availability. Therefore, climate change and hydroclimatic extremes such as droughts can have major impacts on their operations. It is thus important to study the linkage between climate, streamflow, and hydropower generation especially for hydro-dependent power systems in the world. These studies are of major value for effective energy policy making at the water-energy nexus, benefiting both short-term energy supply security and long-term energy transition strategies^6^. These studies require detailed data on hydropower plant and reservoir characteristics, while currently a global dataset with consistent reporting of the information is lacking^6^.

Hydropower plants can be categorized into three main types based on their storage capacity and function: storage (STO), pumped storage (PS), and run-of-river (ROR). ROR is a type of hydropower plant that utilizes the natural flow of rivers and elevation decrease to generate electricity with little or no water storage^7^. Hydropower facilities located on man-made canals or aqueducts are commonly referred to as canal plants. In contrast, storage (STO) hydropower uses large reservoirs that allow them to generate electricity during periods of dry conditions and high energy demand^8,9^. Pumped storage (PS) plants function as energy batteries, with two reservoirs at different elevations. During periods of low energy demand, water is pumped from the lower reservoir to the upper reservoir and then released back to the lower reservoir during high energy demand. The multi-purpose nature of reservoirs and competition for water amongst different sectors and functions (e.g. drinking supply, irrigation, navigation, flood control, and recreation)^10,11^ also impact the outflow from reservoirs and thereby influencing hydropower generation^12,13^. Moreover, the growing demand for water in water-stressed areas–driven by factors such as increased irrigation needs, climate change, industrial usage, and hydropower generation has led to the development of Inter-Basin Transfer (IBT) projects^14^. These projects move water from one basin or river to another, thus not only impact the hydropower generation capacity of donor basin but also that of the receiving basin by altering streamflow patterns^15^. To effectively simulate hydropower generation, especially for large reservoirs and at sub-annual scale, detailed data is required beyond just streamflow. This includes information on reservoir elevation and tailwater elevation (with their difference, commonly known as plant head), exact reservoir location (latitude, longitude), reservoir operation rules and reservoir characteristics such as the area for evaporation losses^16,17^. Furthermore, to effectively simulate hydropower generation in IBT projects, where water transfer is managed manually, it is essential to have data on the project’s water transfer volume capacity, sectoral water demands, and the water allocation strategy^15^.

Previous studies that analysed drought and climate change impacts on existing hydropower highly differ in their levels of detail. Hydropower usable capacity were analysed for 24,515 hydropower plants globally^18,19^, but the head dynamics were disregarded due to lack of detailed data on reservoir characteristics. Turner *et al*.^16^ applied detailed modelling with time-varying head information and evaporation losses from reservoir, but only 1,593 hydropower plants globally were examined due to data limitations. Other studies which used detailed modelling techniques focused either on single river basin^20^ or specific regions such as the United States^21,22^ and Europe^23^.

The primary barrier to detailed global-scale modelling is the lack of a comprehensive, open-access dataset that integrates both hydropower plant characteristics (e.g., installed capacity, hydraulic head, plant type) and reservoir data (dam height, surface area, and volume). Existing hydropower plant datasets differ in their coverage and level of details. For instance, the global power plant dataset from the World Resource Institute (WRI)^24^ provides information on nearly 7,000 existing hydropower plants, including installed capacity, generation, and commissioning year. However, this WRI global hydropower plant datasets lacks reservoir attributes. Regional datasets often offer more details. For example, the recently published Renewable Power Plant database for Africa (RePP)^25^ includes information on both existing and planned hydropower plants in Africa. Together with plant attributes, it provides plant type (ROR, STO and PS), dam height, and reservoir volume for some of the hydropower plants. Similarly, the JRC hydropower database^26^, developed as part of the Water-Energy-Food-Ecosystem-Nexus project provides certain reservoir information such as dam height and reservoir volume. Regardless, both regional datasets lack information on reservoir location, and none of the two datasets provide the actual head of hydropower plants. Based on a comprehensive literature review, the most complete database linking hydropower with reservoirs is, to our knowledge, only publicly available for the United States. The linking of Existing Hydropower Assets (EHA)^27^ dataset with Hydropower Infrastructure - LAkes, Reservoirs, and RIvers (HILARRI)^28^ dataset, both provided by OAK RIDGE National Laboratory, using common id (eha_id), provides not only reservoir characteristics but also reports actual head of each hydropower plant. On the other hand, various reservoir datasets exist, but they usually lack information on hydropower plant characteristics (e.g. plant type, head). The Global Reservoir and Dam (GranD)^29^ database, the most comprehensive reservoir dataset, offers more than 50 attributes on dams and reservoirs. Yet, due to its level of detail, this dataset is limited to 7,320 dams. The recently developed Georeferenced global Dams and Reservoir (GeoDAR)^30^ and GlObal geOreferenced Database of Dams (GOODD)^31^ provide the locations of over 20,000 and 38,000 dams and reservoirs, respectively. However, they do not include additional attributes such dam height and reservoir volume.

In this paper, we present a new comprehensive global dataset of existing hydropower plants by combining open-source hydropower and reservoir datasets. This new dataset, GloHydroRes^32^, provides not only plant attributes such as location, installed capacity but also reservoir attributes such as dam and reservoir location, dam height, reservoir depth, area and volume. GloHydroRes^32^ is created to support a wide range of applications, from academic research to industrial operation and policy design, by offering a rich and reliable data source. It can be used for hydropower planning, water resource management, water-energy nexus and climate impact and adaptation assessment.

**Fig. 1:**
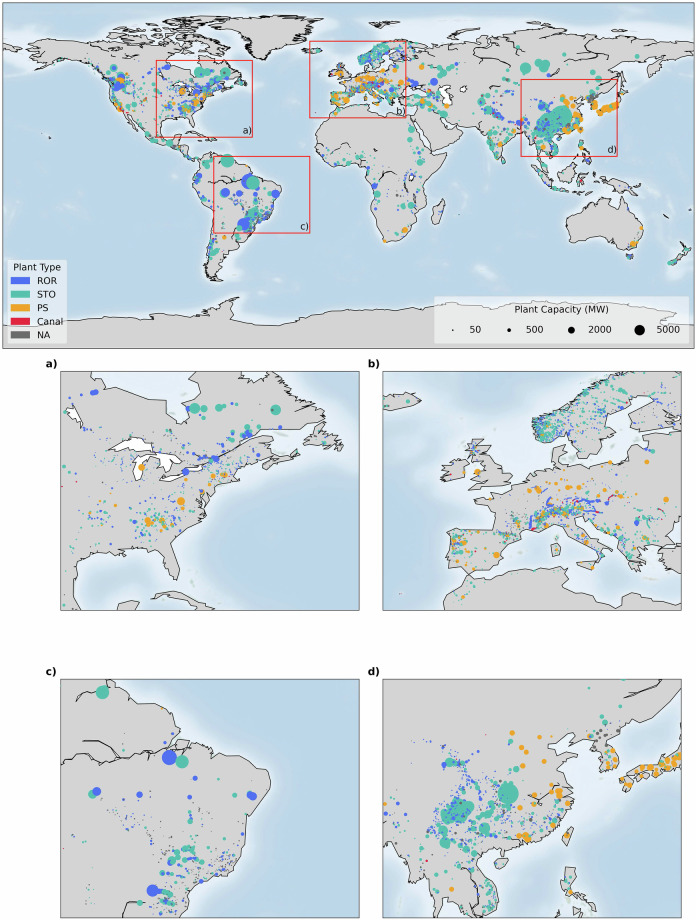
Global spatial distribution of hydropower plants, with color representing the hydropower plant type, i.e. run-of-river (ROR), storage (STO), pumped storage, (PS), Canal or not available (NA). The bubble size indicates the installed capacity of the hydropower plant. The figure also consists of four subplots, each showing a zoomed-in view of regions with a high concentration of hydropower plants, highlighted by red boxes in the global plot.The label inside the each red boxes is corresponds to the respective subplot.

**Fig. 2:**
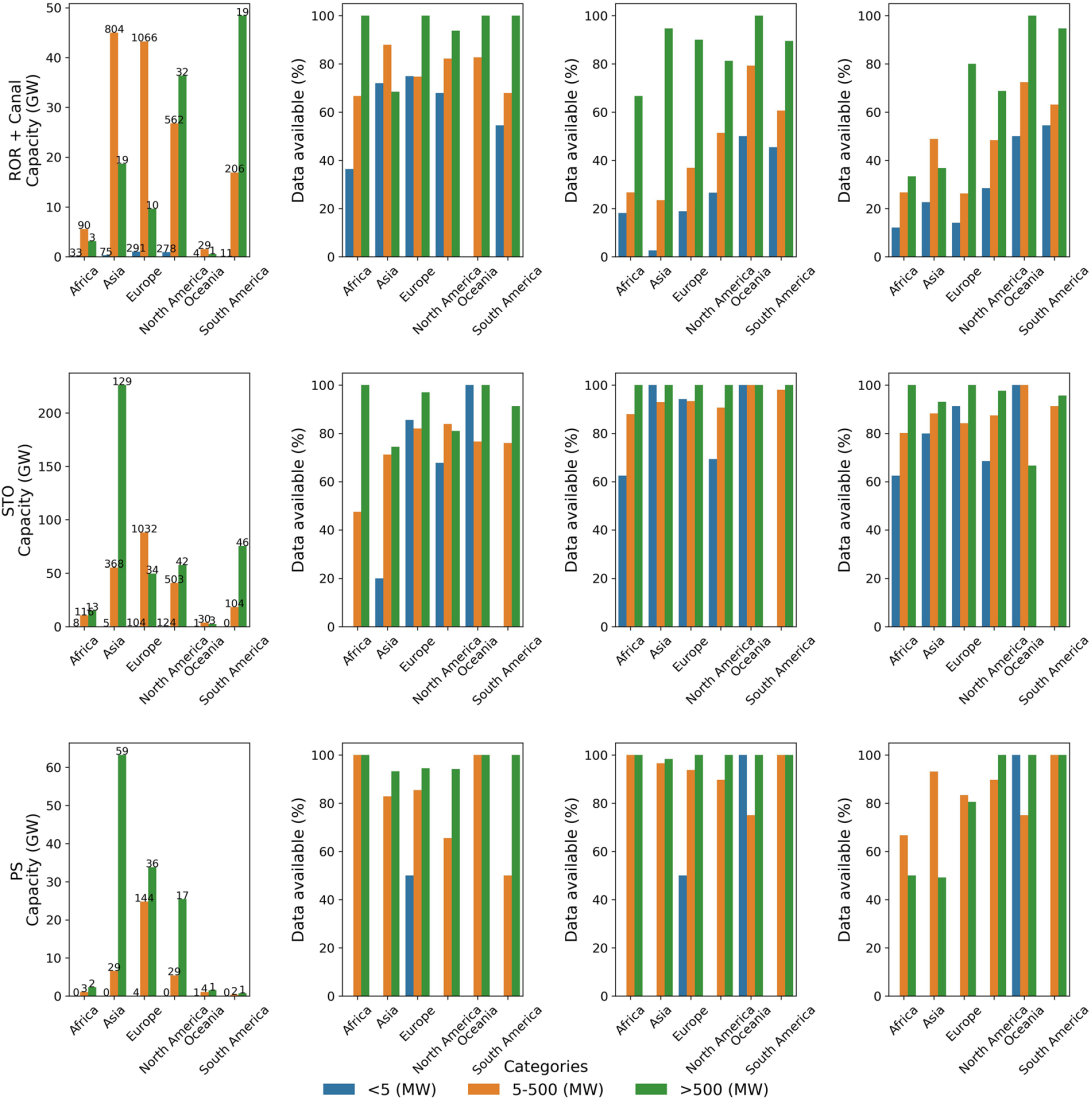
Total installed capacity (first column), along with percentage of data available for head (second column), reservoir volume (third column), and reservoir area (fourth column) in GloHydroRes for different plant types, presented across different continents and, categorized by plant size (<5 MW, 5–500 MW and >500 MW). Reservoir area and volume data are often unavailable for ROR and Canal types, which are grouped together due to their minimal reservoir requirements (first row). Results for storage (STO; second row) and pumped storage (PS; third row) are presented separately. The numbers above the bars in the first column indicate the number of hydropower plants.

GlohydroRes^32^ provides data for 7,775 hydropower plants with a total installed capacity of 1,096.3 GW across 128 countries. This contributes to 79% and 81% of the global installed capacity when compared with installed hydropower data as reported by the U.S. Energy Information Agency (EIA) for 2022^33^ and the International Renewable Energy Agency (IRENA) for 2023^34^ respectively. In Europe, data is available for 2,888 plants, followed by North America and Asia with 1,880 and 1,823 plants respectively. However, in terms of installed capacity, Asia leads with 474.1 GW being represented, followed by Europe with 205.3 GW and North America with 199.44 GW. This is not only due to the presence of large hydropower plants in Asia, such as the Three Georges Dam (22,500 MW) (Fig. 1), but also because data for smaller hydropower plants are more readily available in Europe and North America compared to Asia (e.g. Figure 2). Out of these 7,775 plants, 3,237 (41.6%) are ROR plants and 2,658 (34.2%) are STO (e.g. Table 1). Only 4.3% classified as PS (Table 1) and most of these are located in Asia, Europe, and North America (Fig. 2). Regarding data availability, attributes such as head, reservoir area and volume of large hydropower plants (i.e. installed capacity > 500 MW) are typically more easily accessible across all continents. Data for smaller hydropower plants (i.e. installed capacity < 5 MW) are generally less well represented, but their total contribution to the overall installed capacity is small (0.3%) (Fig. 2). Furthermore, 170 hydropower plant have been identified as being impacted by IBT projects. These plants are located either on the IBT projects themselves or upstream and downstream of them.

**Table 1:** Table represents the number of hydropower plants, their installed capacity, and the coverage for each type of hydropower plants, i.e. run-of-river (ROR), storage (STO), pumped storage, (PS), Canal or not available (NA).

Type	Number of hydropower plants	Capacity (GW)	Coverage (%)
**Global**			
ROR	3,237	248.5	41.6
STO	2,658	638.3	34.1
PS	332	166.3	4.2
Canal	297	9.7	3.8
NA	1,251	33.2	16
**Asia**			
ROR	816	66.6	47.6
STO	551	317.8	30.2
PS	91	71.3	4.9
Canal	55	2.5	3
NA	258	15.6	14.1
**Africa**			
ROR	121	8.7	42.3
STO	137	25.5	47.9
PS	5	3.3	1.7
Canal	5	0.05	1.7
NA	18	0.5	6.2
**North America**			
ROR	748	65.2	39.7
STO	669	98.8	35.5
PS	46	30.8	2.4
Canal	124	4	6.5
NA	293	5.6	15.5
**South America**			
ROR	228	65.2	28.6
STO	148	93.5	18.6
PS	3	1	0.3
Canal	9	0.2	1.1
NA	408	6.2	51.2
**Europe**			
ROR	1,240	45.8	42.9
STO	1,119	96.5	38.7
PS	181	57	6.2
Canal	102	2.8	3.5
NA	246	2.9	8.5
**Oceania**			
ROR	32	2	31.3
STO	34	6	33.3
PS	6	2.5	5.8
Canal	2	0.03	1.9
NA	28	2.2	27.4

Coverage represents the proportion of the total number of hydropower plants for each type.

## Methods

**Fig. 3:**
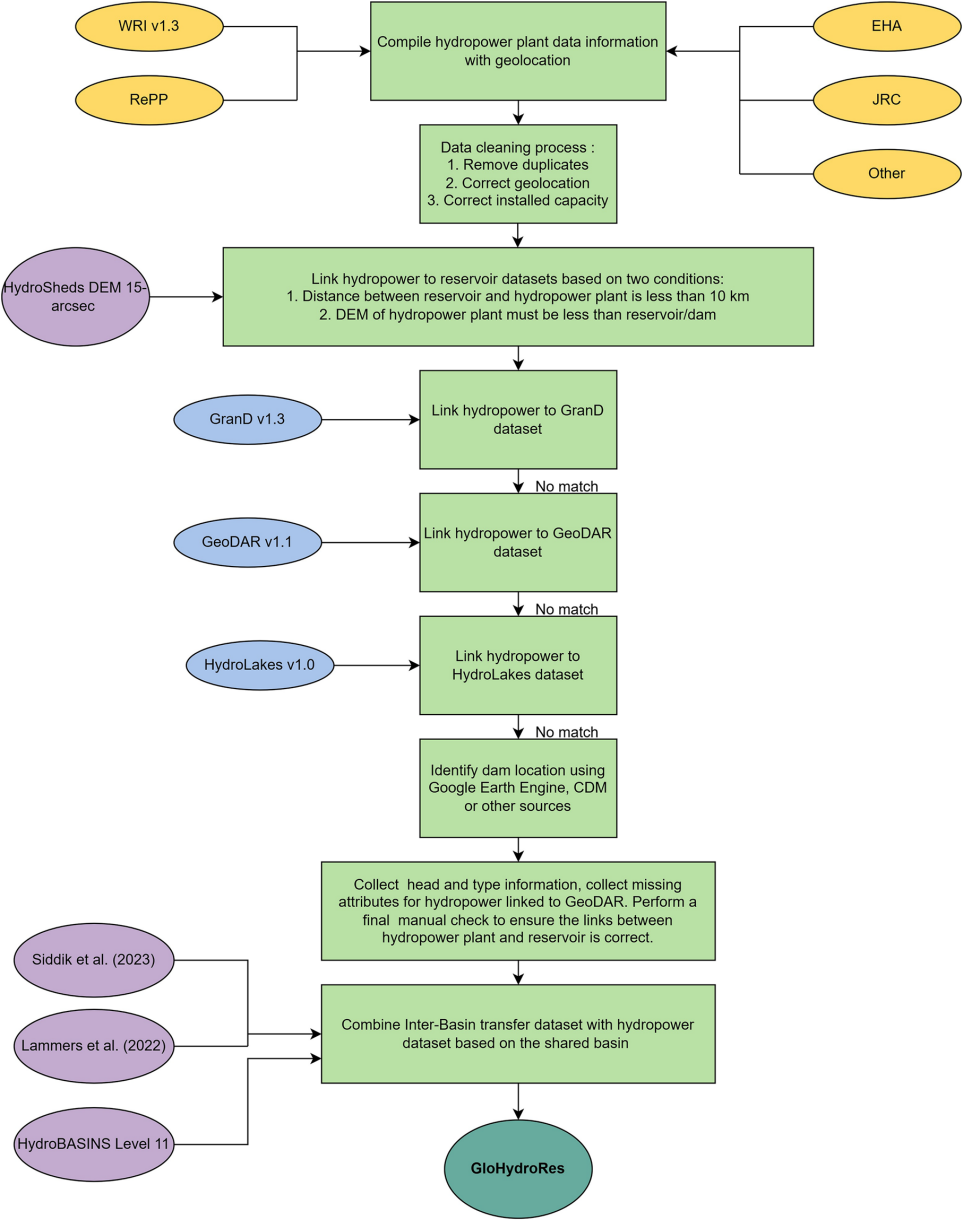
Flow chart illustrating the main steps taken in developing the GloHydroRes dataset. The hydropower datasets used include the World Resource Institute v1.3 (WRI), the Renewable Power Plant database (RePP), the Joint Research Commission (JRC), the Existing Hydropower Assets (EHA), as well as other sources such as research articles and the Open Infrastructure Map. Reservoir datasets utilized are the Global Reservoir and Dam v1.3 (GranD), the Georeferenced global Dams and Reservoir v1.1 (GeoDAR) and HydroLAKES v1.0.

An overview of the datasets used and steps followed to develop the GloHydroRes^32^ dataset is provided in the flowchart in Fig. 3. Details about the attributes collected are presented in Table 2 and are further explained in the following subsections.

**Table 2:** Attributes collected in the GloHydroRes dataset, their brief descriptions, units and the global coverage (%).

Attributes Name	Description	Units	Global Coverage (%)
ID	Unique ID		100
country	Country Name		100
name	Plant name		100
capacity_mw	Installed capacity of hydropower plant	MW	100
plant_lat	Latitude of hydropower plant (WGS)	Degree Decimal (°)	99.9
plant_lon	Longitude of hydropower plant (WGS)	Degree Decimal (°)	99.9
plant_type	Type of hydropower plant	ROR/STO/PS/Canal/NA	83.9
plant_type_source	Source which provided plant type information		79.6
year	Power plant first started operation and if not available then commissioned year		91.4
plant_source	Source (dataset) which provide hydropower plant attributes.	WRI/RePP/EHA/Other (e.g. Google Earth Engine, OpenStreetMap, research articles link)	100
plant_source_id	Unique ID of the source dataset for hydropower plant information. Following column of each dataset was used as unique id: “gppd_idnr” in WRI, “ID” in RePP, “id” in JRC and “eha_ptid” in EHA. Not applicable if information collected during google search.		99.1
dam_name	Name of dam		52.2
dam_height_m	Height of dam	Meters (m)	50.3
dam_height_source	Source which provided dam height		50.3
res_name	Name of reservoir		18.4
res_dam_source	Source which provided dam/reservoir geolocation. If source is not GranD, GeoDAR or if source is not GranD, GeoDAR or HydroLAKES then dam/reservoir geolocation may be available in man_dam_lat and man_dam_lon.	GranD, GeoDAR, HydroLAKES, Other (e.g. Google Earth Engine, CDM document link)	89.5
res_dam_source_id	Unique ID of the source dataset which reservoir linked to corresponding hydropower. Following columns were used as unique id: “grand_id” in GranD, “id_v11” in GeoDAR and “Hylak_id” in HydroLAKES. Not applicable if collected from Google Search Engine, Clean Development Mechanism document or other sources.		58.5
man_dam_lat	Latitude coordinate of dam (WGS84) with manual search	Decimal degree (◦)	31.4
man_dam_lon	Longitude coordinate of dam (WGS84) with manual search	Decimal degree (◦)	31.4
river	Name of river		81.9
head_m	Reported head	Meters (m)	68.8
head_source	Source which provided head information		68.8
res_avg_depth_m	Reservoir depth	Meters (m)	47.7
res_area_km^2^	Reservoir area	Square kilometre (km^2^)	56.3
res_vol_km^3^	Reservoir volume	Cubic kilometre (km^3^)	60.5
res_attr_source	Name of source which provided reservoir volume/area/depth information.		65.8
res_attr_id	Unique ID of the source dataset which provided reservoir attributes (volume, area, and depth). Not applicable if collected from more than one source or google search		49.5
hydrolakes_id	HydroLAKES ID		43.6
final_comments	Final Comments		

### Data collection

The GloHydroRes^32^ dataset was built by compiling publicly available datasets on existing hydropower plants and reservoirs with creative common license. The following procedure was used to create the GloHydroRes dataset. First, data on hydropower plants were compiled from the WRI^24^, EHA^27^, RePP^25^ and JRC^26^ datasets. While datasets like RePP^25^ also include information on planned hydropower projects, this data was not incorporated due to the lack of detailed information about their plant and reservoir characteristics. During compilation process, duplicate entries were encountered because hydropower plants in the global WRI dataset were also present in the regional RePP and JRC datasets. These duplicates were removed by manually comparing the locations and installed capacities of the hydropower plants, retaining entries from the regional datasets (RePP, JRC, and EHA) as they provide more detailed information compared to the global WRI dataset. In the second step, reservoirs were linked to their corresponding hydropower plants using the GranD^29^, GeoDAR^30^ and HydroLAKES^35^ datasets. These datasets were chosen due to greater accuracy of reservoir shapes, compared to other datasets which often use simplified rectangular representations. GDAT^36^ data was also used to fill in the reservoir attribute missing in GeoDAR and HydroLAKES datasets. It is important note that the HILARRI dataset developed for the United States already links hydropower with reservoir data from GranD and the National Inventory of Dams (2021)^28^. However, to reduce number of sources of reservoir information, we retained the hydropower plants linked with GranD and connected the remaining plants with either GeoDAR or HydroLAKES. Furthermore, reported head and hydropower type (ROR, STO, Canal, and PS) were manually collected from various sources, including turbine supplier companies, power utility companies, research articles, energy agencies, Clean Development Mechanism (CDM) documents from United Nations Framework Conventions on Climate Change (UNFCCC)^37^ and websites such as power technology^38^ and Wikipedia^39^. It is important to note that the hydropower plant types in the GloHydroRes^32^ dataset is derived from original hydropower data sources such as RePP for Africa^25^ and JRC for Europe^26^, following the decision tree (Fig. 3). For the United States, plant type information is obtained from the Hydropower Reform Coalition^40^. Similarly, data for South America, Asia and Oceania continent are compiled from diverse sources such as Clean Development Mechanism (CDM)^37^, websites like power technology^38^. We prioritize using the categorization provided by the original sources rather than using arbitrary thresholds to identify hydropower plant types, which might be subject to the assumptions and choices of the thresholds. While collecting head attribute, we encountered significant discrepancies in how head values are reported by different sources with a range of terminologies, including gross, net, nominal, hydraulic, effective, rated, maximum, minimum, and average head. The gross head represents the difference between headwater and tailwater elevations, while the net head also accounts for friction losses in the penstock and losses in the conveyance system^41^. Previous studies have highlighted the importance of using net head to simulate hydropower generation accurately^41–43^. Rated and nominal head are often provided by turbine manufactures to represent the optimal operating condition for their turbines. To ensure consistency, we prioritize using net head followed by rated, nominal and gross head. When head values are reported as maximum, minimum or average, we gave priority to the average head, followed by maximum and then minimum head. For the United States, the EHA reports the rated head of all hydropower plants^27^. Therefore, these head values were directly incorporated into GloHydroRes^32^. During the Google search for information on head and hydropower type attributes, existing or new hydropower plants were found that were not present in any existing datasets. These hydropower plants were incorporated into GloHydroRes^32^, geolocated using either Google Earth Engine or Open Street Map, and linked to the corresponding reservoir. Hydropower projects located on IBT projects were identified based on shared basins between the hydropower projects and the IBT projects. For this purpose, two IBT datasets were used: Siddik *et al*.^44^ (for the United States and Canada) and Lammers *et al*.^45^ (global coverage). The HydroBasin^46^ dataset was utilized for basin information. Attributes related to IBT projects–such as project name, start year, status, water flow direction, minimum, maximum, and mean discharge flow for project, as well as project length (Table 3)–are provided in separate file. This file can be linked with GloHydroRes dataset using the common identifier glohydrores_id corresponding to the ID in GloHydroRes^32^.

**Table 3:** Attributes collected for inter-basin transfer (IBT) projects associated with hydropower plants in GloHydroRes.

Attributes Name	Description	Units
glohydrores_id	Unique ID of GloHydroRes dataset	
project_name	Name of IBT project	
hybas_id	Unique ID of basin from HydroBasin	
ibt_source	Source of IBT data	
ibt_id	Unique ID of the source dataset. Following columns were used as unique ID: “Link ID”: Siddik *et al*. (2023) and “ID”: Global Inter Basin Hydrological Transfer Database (Lammers *et al*. 2022)	
ibt_flow_direction	Inflow: reservoir receives water from IBT project, Outflow: reservoir supply water to IBT project, Inflow/Outflow: Hydropower plant at intermediate location, Indirect: Hydropower upstream/downstream, indirectly impact IBT	
ibt_from	Location where the IBT project originates	
ibt_to	Location where the IBT project terminates	
ibt_status	Status of IBT project: Completed, Under Construction or Proposed	
ibt_startyear	Start year of IBT project	
ibt_minflow	Minimum flow in the IBT project	Cubic meter per day (m^3^/day)
ibt_maxflow	Maximum flow in the IBT project	Cubic meter per day (m^3^/day)
ibt_meanflow	Mean flow in the IBT project	Cubic meter per day (m^3^/day)
ibt_lengthtrasnfer	Total length of transfer	Kilometre (Km)
ibt_link	commented about how hydropower/reservoir uses IBT water	
ibt_comment	IBT regarding comment	

### Data processing

We used two conditions to assign a hydropower plant to its nearest reservoir (1) distance between the hydropower plant and dam must be less than 10 kilometres (proximity criteria) and (2) elevation of the hydropower plant must be lower than elevation of dam (elevation difference criteria). The global digital elevation model (15 arc-seconds resolution) from Hydrosheds^47^ was used to obtain elevation estimates for both hydropower plants and dams. If multiple dams met both the proximity and elevation difference criteria in a single reservoir dataset, then nearest dam was assigned to that hydropower plant. First hydropower plants were matched with GranD dataset and if no reservoir matches both conditions, then GeoDAR dataset was used. GranD was given priority over GeoDAR because it provides a higher level of detail. Since GeoDAR provides only the geolocation of dams and reservoirs without attributes such as dam height, river name, reservoir area and volume, this information was supplemented from other sources. These sources include reservoir datasets HydroLAKES, GDAT or were manually incorporated from e.g. research articles (Fig. 3). Furthermore, if neither of the reservoir datasets (i.e. GranD and GeoDAR) contained a reservoir following both conditions, then HydroLAKES data was used. It is important to note that although HydroLAKES provides information on 1.43 million waterbodies, it was given priority after GranD and GeoDAR. This decision was made because HydroLAKES is a dataset of lakes rather than reservoirs. Using HydroLAKES can introduce uncertainties, as there is an increased probability that a small nearby lake rather than a large, purpose-built reservoir is being unintendedly assigned to a hydropower plant. If none of the three sources contained reservoir information, a manual search was conducted to identify the hydropower plant reservoir. The dam’s longitude and latitude were then manually obtained from scientific literature or Google Earth Engine search, and this information is displayed in the man_dam_lat and man_dam_lon columns (Table 2). For PS hydropower, only the attributes of the upper reservoir are provided. After all hydropower plants were linked to their nearest reservoirs, a through manual verification process was undertaken. This involved cross-referencing research articles, hydropower utility companies, Google Earth Engine or OpenStreetMap to ensure that all hydropower plants were correctly linked to their respective reservoirs. To ensure traceability between GloHydroRes and the source datasets of hydropower plants and reservoirs, we included the unique ID from the source datasets along with the dataset name^32^. These are shown in the attributes plant_source_id and plant_source for hydropower plants, and res_dam_source_id and res_dam_source for reservoirs in Table 2. For example, if the reservoir of a hydropower plant matched with one of the GranD dataset reservoirs, then we provided the corresponding “grand_id” in the res_dam_source_id column and “GranD” value in the res_dam_source column (Table 2). This will allow future users to link to the original datasets to update and add relevant new information which is not available in the GloHydroRes^32^ but provided by the original dataset. Similarly, IBT attributes include ibt_id and ibt_source to capture additional attributes provided by the original source (Table 3).

## Data Records

GloHydroRes^32^ is available in Excel (.xlsx) and Comma-Separated Values (.csv) formats and can be accessed through the open-source platform Zenodo at 10.5281/zenodo.14526360 under the names GloHydroRes_vs1.xlsx and GloHydroRes_vs1.csv. The excel file contains four worksheets: “Summary”, “Acronyms”, “Field Description” and “Data”. The “Summary” worksheet offers brief overview of GloHydroRes, while “Acronyms” explains the abbreviation used in dataset. The “Field Description” contains details about collected attributes and the “Data” worksheet contains the actual data^32^. The CSV format includes only the core dataset from the “Data” worksheet along with a README file for guidance. A total of 29 attributes were collected (Table 2) covering both plant and reservoir characteristics from various aspects. Plant attributes include plant name, installed capacity, plant geolocation, and type. Reservoir attributes include dam height, dam geolocation, river name, reservoir depth, area, and volume. Similarly, the file IBT_GloHydroRes_Hydropower_Combined.xlsx and IBT_GloHydroRes_Hydropower_Combined.csv contains information about GloHydroRes hydropower projects located on inter-basin transfer projects. Overall, the file includes 15 attributes related to inter-basin transfer and can be linked to GloHydroRes using the glohydrores_id column in the IBT_GloHydroRes_Hydropower_Combined.xlsx file and ID column in the GloHydroRes_vs1.xlsx file^32^. All geolocations are provided in the World Geodetic System (WGS) 84 coordinate system. GloHydroRes^32^ is an open-source dataset developed by combining other open-source hydropower and reservoir datasets. The code used to generate the GloHydroRes dataset (see the “Code Availability” section} is also open accessible to everyone. Consequently, anyone can contribute to updating and enhancing the dataset in the future when new hydropower plant and reservoir data will become available.

## Technical Validation

### Data availability review

**Fig. 4:**
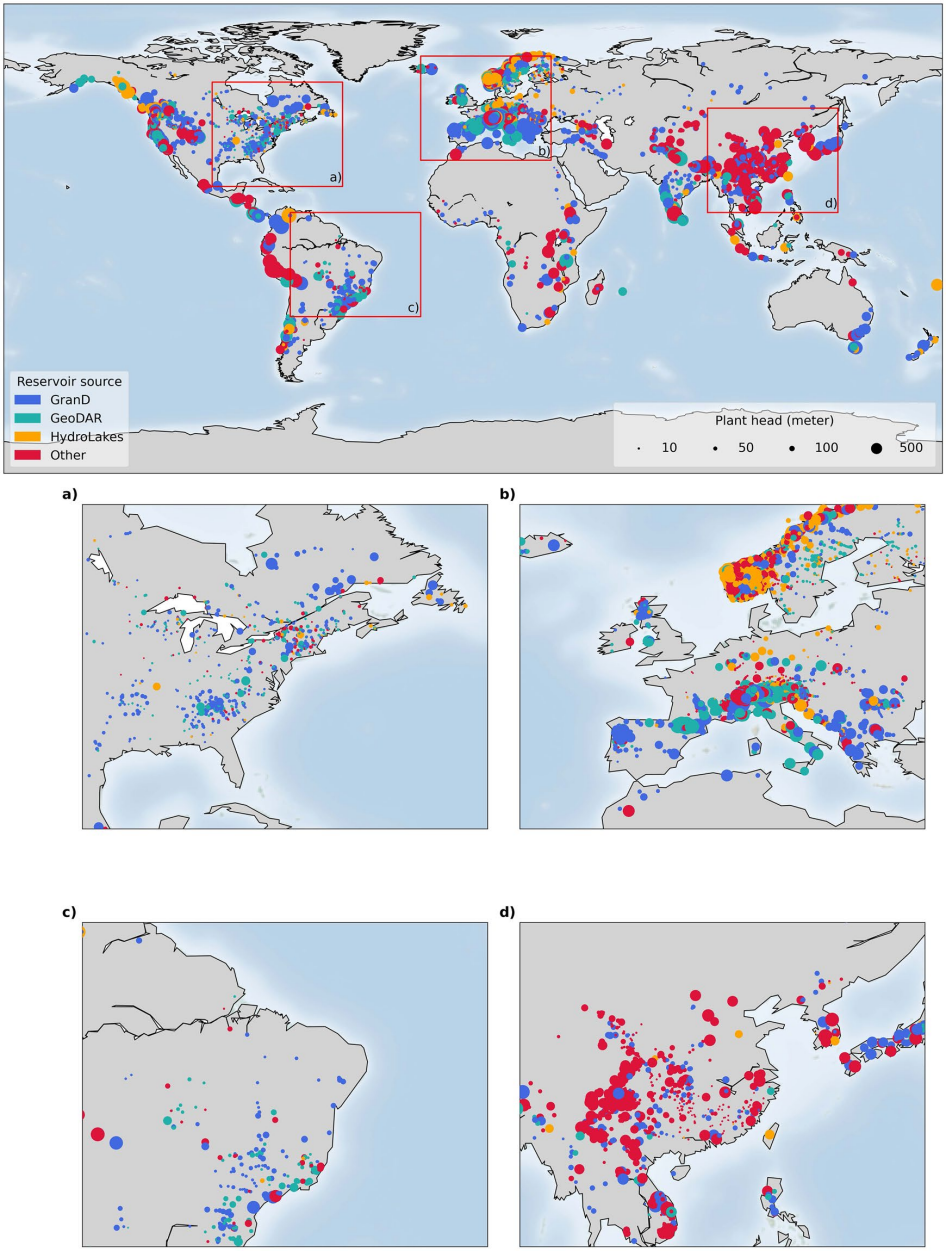
Global spatial distribution of hydropower plants, with colour representing the source of the reservoir dataset and bubble size indicating the hydropower plant head. Zoomed-in view of four regions are provided, highlighted by red boxes in the global plot, with labels inside the boxes corresponding to their respective regional plots.

GloHydroRes^32^ expands regional datasets, such as HILARRI^28^, and provides the first comprehensive global dataset that covers both hydropower plant attributes and reservoir characteristics. In total, 4,552 hydropower reservoirs were identified from sources like GranD, GeoDAR or HydroLAKES (Table 4). Additionally, 2,407 hydropower reservoirs were identified through manual research, with 537 in China (Table 4 & Fig. 4). Moreover, 5,353 hydropower head data were provided, compared to just 971 offered by Wan *et al*.^48^ or those found only in regional datasets such as EHA^27^. To ensure the quality of the dataset, all the attributes of dataset were verified. ArcGIS Pro^49^ was used for manual verification to ensure the data accuracy in linking hydropower with reservoirs.

**Table 4:** The table provides details on the number of hydropower plants, their total capacity (in GW), and the share (in percentage) for each hydropower dataset.

Group	Source	Number of hydropower plants	Capacity (GW)	Share (%)
Hydropower	WRI	3,983	794.3	51.2
	RePP	242	27.7	3.1
	JRC	2,263	164.2	29.1
	EHA	1,223	101.7	15.7
	Other(e.g. Open Infrastructure Map)	64	8.2	0.8
Reservoir	GranD	2,301	716.4	29.5
	GeoDAR	1,496	144.2	19.2
	HydroLAKES	755	44.2	9.7
	Other (e.g. Google Earth Engine)	2,407	174.7	30.9
	NA	816	16.6	10.4

For the reservoir group, it highlights the contribution of each dataset in linking hydropower plants with their respective reservoirs. Share represents the percentage contributed by each specific dataset to the total number of hydropower plants. “NA” in the reservoir group indicates hydropower plants for which reservoir and dam locations are not available.

To evaluate completeness of GloHydroRes^32^, country-level aggregated installed capacity is compared with data provided by international intergovernmental organisation, the International Renewable Energy Agency (IRENA)^50^ and the International Energy Agency (IEA)^51^ and the U.S. Energy Information Administration (EIA)^52^. These agencies collect data through partnerships with governmental organizations in each country, and therefore can be considered to cover nearly all installed hydropower capacity within a country.

**Fig. 5:**
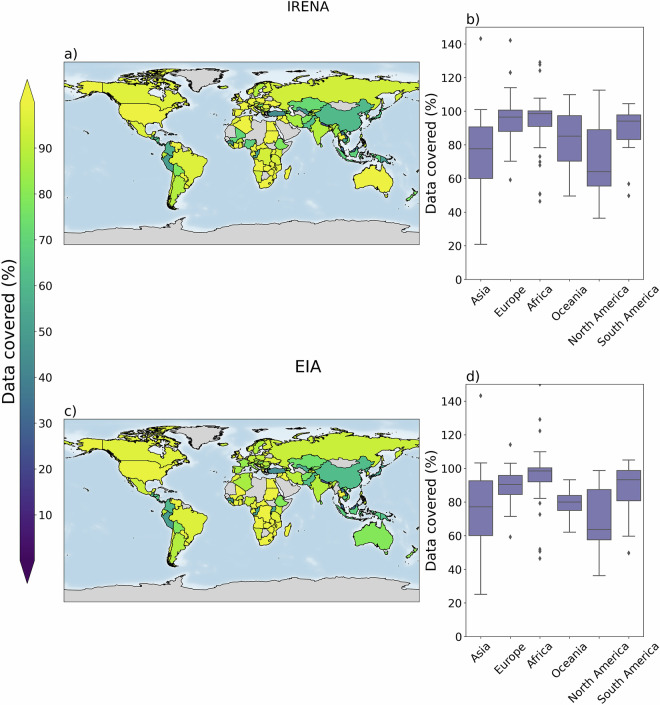
The total installed capacity in GloHydroRes is compared at country level with IRENA data for 2023 and EIA data for 2022. The left panel shows the spatial distribution of the percentage contribution in installed capacity at country level covered by GloHydroRes compared to IRENA and EIA. The right panel presents boxplots representing the distribution of the proportion of installed capacity covered for countries in each continent, comparing GloHydroRes with IRENA and EIA.

GloHydroRes^32^ installed capacity was compared with IRENA data of 2023^34^ and EIA data of 2022^33^. IEA data was not used for comparison as it is not a freely available dataset and requires a paid subscription^53^. In comparison that follow, data from IRENA is presented without bracket, while data from the EIA is shown in brackets. European countries are well-represented in GloHydroRes, with half of the countries in these continents having more than 96% (90%) of their installed capacity covered, compared to reported estimates of IRENA (EIA) data (Fig. 5). Similarly, African countries have 98% of their installed capacity covered, compared to both the IRENA and EIA data. African countries, such as Angola, Cameroon, Egypt and South Africa, the installed capacity even exceeds the figures reported by IRENA and EIA (Fig. 5). Similarly, in European countries like Austria, Croatia, France, Spain, and Switzerland, installed capacities surpass those reported by IRENA, while in Lithuania and United Kingdom, they exceed EIA estimates. This discrepancy can be attributed to the use of not only the global WRI dataset but also the detailed regional datasets provided by JRC for Europe and RePP for Africa. These additional datasets ensure that smaller hydropower plants, which may be missing from the IRENA and EIA data, are well represented. In contrast, the representation of several North American countries such as Costa Rica, Honduras, and Panama is low with only 55% (55%), 56% (60%) and 57% (59%) of installed capacity covered in GloHydroRes, respectively, compared to IRENA (EIA). This is because the hydropower plant data for these countries come solely from the WRI dataset, and no regional or country-specific dataset is available. The WRI dataset typically includes information on hydropower plants installed before 2019, thus missing information on recent installations. However, other North American countries, like the United States and Canada, benefit from extensive data availability, leading to a more comprehensive picture in GloHydroRes. South American countries exhibit wide variability with Bolivia, Colombia and Peru having below 65% (60%) representation, while Brazil, Paraguay, Uruguay, and Venezuela have more than 95% (95%) representation in terms of installed capacity when compared to IRENA (EIA) data. Asian countries have a moderate level of representation, with a median coverage of 77% (77%). Interestingly, in some countries, EIA estimates of installed capacity for 2022 are higher than the IRENA estimates for 2023 (Fig. 5). This may be due to difference in the data sources used by each agency within individual countries and variations in their data collection methodologies. Overall countries or continents with dedicated hydropower datasets (e.g. JRC for Europe, RePP for Africa, and EHA for the United States) tend to have better representation in GloHydroRes compared to other regions.

It is important to note that GloHydroRes^32^ has information on 7,775 hydropower plants, compared to the 24,515 hydropower plants included in the study by Van Vliet *et al*.^18^, which evaluated the impact of climate change and droughts on hydropower. Due to limited resources and the extensive time required, it is not feasible to obtain information on all hydropower plants in a country, particularly smaller plants (less than < 5 MW), as covered by the Platts dataset^54^ used by Van Vliet *et al*.^18^, which is provided by S& P Global, an organization with significant resources. However, a key advantage of GloHydroRes^54^ is that it is freely accessible, unlike the Platts^54^ dataset, which requires a license. Additionally, GloHydroRes provides more detailed attributes, such as reservoir characteristics, compared to Platts^18^.

### Validating hydropower generation

Hydropower production at a turbine is usually simulated using the following equation.

$${P}_{t}=\rho \ast g\ast h\ast {q}_{t}\ast \eta $$

where P_t_ is hydropower generated (kW), ⍴ is density of water (1000 kg m^−3^), g is gravitational acceleration (9.81 m s^−2^), h is head (m), q is streamflow (m^3^ s^−1^) and η is turbine efficiency. Due to the lack of actual or reported head data, previous studies often use proxies such as the elevation difference between reservoir and turbine location^55^, dam height^18^ or reservoir level^16^. However, these methods can overestimate generation if the turbine is not located at the dam toe or underestimate if the turbine is located underground. We validate this by simulating monthly hydropower generation for four hydropower plants: Crystal dam (31.5 MW) on the Gunnison River, Wanapum Dam (1,098 MW) on the Columbia River in the United States, Estreito (1,087 MW) on Tocantins River and Jupiá (1,551 MW) on the Parana River in Brazil. We selected these plants because they are of the ROR type and do not have large reservoirs, thus eliminating the need to simulate reservoir operations which can impacts the monthly hydropower generation^56^. Additionally, these hydropower plant sites were selected due to the availability of observed streamflow data close to these hydropower stations. Here we used dam height and reported head data from GloHydroRes^32^ as the head parameter in the above mentioned equation to calculate the monthly hydropower generation and compared the results with actual generation data obtained from Turner *et al*.^56^ for the United States and Operdor Nacional do Sistema Eletrico (ONS)^57^ for Brazil. Monthly observed streamflow data were obtained from Global Runoff Data Center (GRDC)^58^ for the United States and Catchment Attributes and Meteorology for Large-sample Studies (CAMELS) dataset for Brazil^59^. Note that, since information regarding turbine efficiency (η) is generally unavailable, a default value of 1 is assumed.

**Fig. 6:**
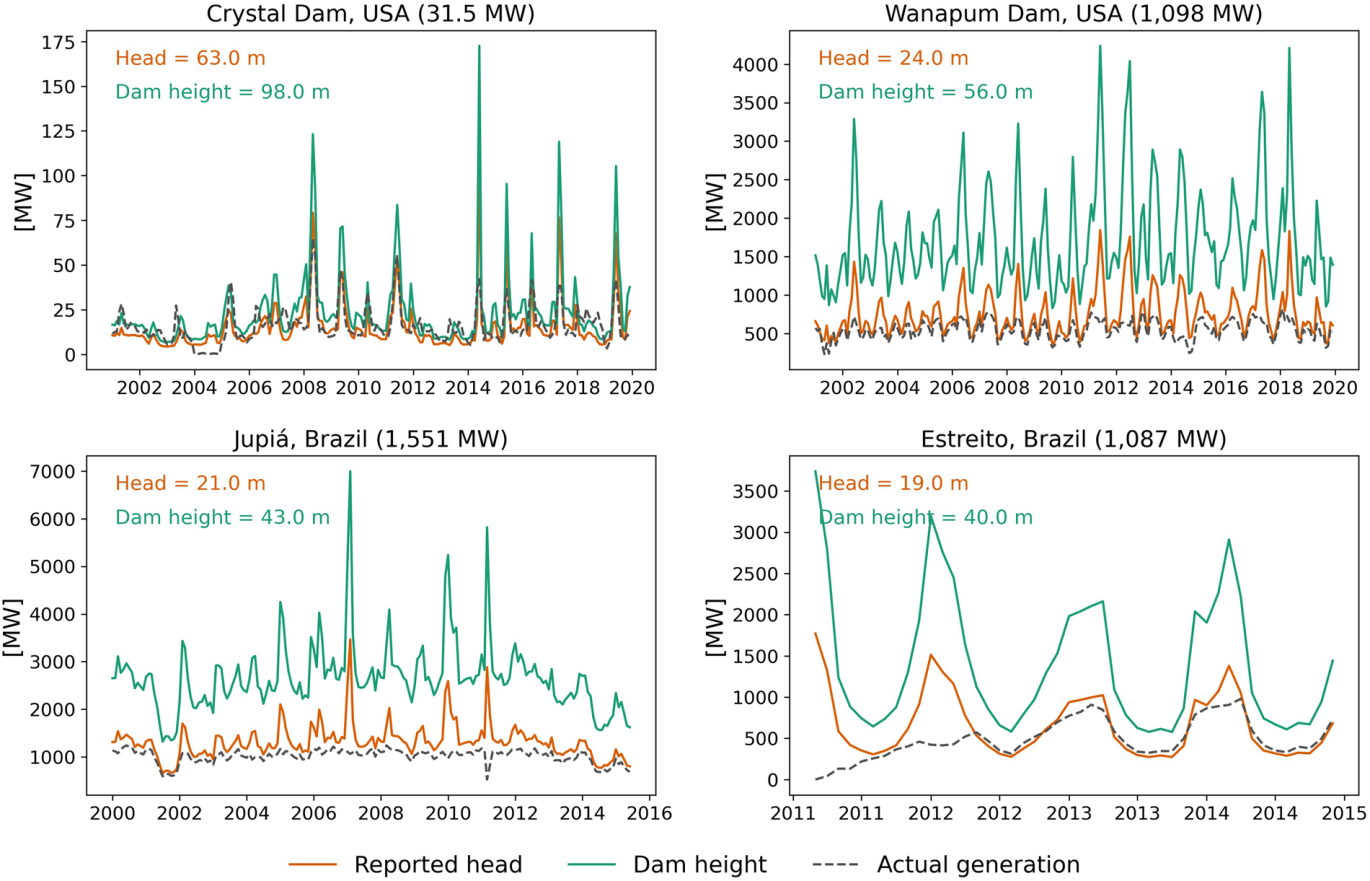
The monthly generation of four hydropower plants is simulated using head as (1) dam height and (2) reported head from the GloHydroRes dataset, applying the physical based equation: ${{\rm{P}}}_{{\rm{t}}}={\rm{\rho }}\ast {\rm{g}}\ast {\rm{h}}\ast {{\rm{q}}}_{{\rm{t}}}\ast {\rm{\eta }}$ where P_t_ is hydropower generation, ${\rm{\rho }}$ is density of water (1000 kg m^−3^), g is gravitational acceleration (9.81 m s^−2^), h is head (m), q is streamflow (m^3^ s^−1^) and η is turbine efficiency. The simulated generation is compared with actual monthly generation data. The actual generation data is obtained from Turner *et al*.^56^ for the United States and Operdor Nacional do Sistema Eletrico (ONS)^57^ for Brazil.

The results show that simulated generation using reported head follows actual generation more closely compared to the simulation using dam height (Fig. 6). This finding underscores the potential for enhancing hydropower generation simulations at individual plant sites by considering the detailed head information provided in the GloHydroRes dataset. Despite the improvement gained from using actual head data, simulated hydropower generation does not precisely match actual generation, particularly at the Wanapum and Jupiá sites (Fig. 6). This discrepancy can be attributed to water spill from hydropower plants, which occurs in both controllable and uncontrollable forms. Controllable spill usually occurs in STO plants, which are often mandated by environmental regulations. These regulations require the release of a certain amount of water to maintain downstream water levels for purposes such as fishing, water quality maintenance, ecosystem preservation and accommodation of other downstream uses^6,56,60^. In contrast, ROR plants, are more susceptible to uncontrollable spill, especially during period of high streamflow. In these scenarios, excess water flow exceeds the plant generation capacity, leading to spillover that cannot be harnessed for energy production^61^. This spill is commonly referred to as “non-power spill”. However, site-specific data on spill is generally not available. Notably, it is recognized that ROR plants have typically experience greater non-power spill compared to STO plants^61^. Since the GloHydroRes^32^ dataset provides information on plant types, future studies can leverage this data to estimate non-powered spill based on plant type, enabling more accurate simulations of hydropower generation. Overall, this new open-source dataset can provide crucial information to improve hydropower generation modelling and can support informed decision-making in energy security and planning at the plant level in regions worldwide.

## Data Availability

The Python code used to link hydropower plants to their corresponding reservoirs and to plot figures is available on GitHub (https://github.com/SustainableWaterSystems/GloHydroRes/tree/main).
